# Research on the quality hospice care of elderly cancer patients in China under social work intervention

**DOI:** 10.1186/s12199-020-00867-4

**Published:** 2020-08-01

**Authors:** Li Wang, Zhizhong Wang

**Affiliations:** Deparment of Social Work, Shanxi Medical University, Taiyuan, 030001 China

**Keywords:** Quality hospice care, Elderly cancer patients, Social work intervention

## Abstract

**Background:**

Few studies have specifically addressed quality of life issues for elderly hospice patients. The purpose of this study is to explore various factors and service patterns of the quality of life of end-of-life care for the elderly.

**Methods:**

We collect the data and make small-scale exploratory study via semi-structured individual interviews. Data were collected from the family of 2 elderly cancer patients receiving hospice services, and the data were analyzed qualitatively.

**Results:**

After investigation, we found that elderly people in hospice care, regardless of age, are suffering from physical and psychological pain and do not want to spend the rest of their lives in the hospital, but want to die in their own homes.

**Conclusions:**

Both hospitalization and in-home care can improve resource utilization, and the key is to find various factors affecting the quality of life. Improving the quality of life is what patients and their families need most.

## Introduction

The hospice care movement emphasizes an integrated approach that strives to focus on the personal life experience and lays stress on the importance of multidisciplinary collaboration since the mid-1960s. The purpose of this study is to obtain the most authentic, direct, and original data from multiple aspects such as cognition, experience, and behavior from the perspective of the real experience of medical staff and social workers in the process of caring for the dying patients’ loss of life, summarize the implementation status of hospice care, analyze the problems encountered and give reasonable explanations, to improve the quality of life of end-of-life care patients through the efforts of a multidisciplinary team of hospice care, give full play to professional advantages to safeguard the rights and dignity of end-of-life care patients, and to enable them to walk the last stop of their life journey with dignity and peace. The intervention of social work methods is very important, so it has a very close relationship with social work practice. Death is an essential part of life, so it certainly affects each member of society. The national cancer center released the latest cancer data from China, showing that 4.29 million new cases of cancer are reported annually in China, accounting for 20% of new cases worldwide and 2.82 million deaths, and the elderly, in particular, were 36%. The goal of quality hospice care is not to cure the disease, but to minimize the suffering and pain of the patient [1]. According to the statistics of China cancer registration 2017, the incidence of cancer among the elderly in our country exceeds 75%.

There are different approaches to the field of hospice care. Retrospective design study is a good research method that analyzes the reliability and validity issues associated with a follow-back design. Although methodological challenges beset both prospective and retrospective data collection, use of both methods in combination or in sequence provides a clearer understanding of the complex, multidimensional issues involved in providing care to dying individuals [2]. Experimental method is a very effective research method, and it was divided into a coordination group and a control group. This coordinating service made little difference to patient or family outcomes, perhaps because the service did not have a budget with which it could obtain services or because the professional skills of the nurse coordinators may have conflicted with the requirements of the coordinating role [3]. Random sampling is considered to be one of the most representative research methods. The survey has been successful, both in providing districts with information on local services, and in obtaining a large, broadly representative, sample of deaths, thus enabling many outstanding questions about the needs of dying patients and their families, and about appropriate service provision, to be addressed [4].

Hospice care institutions in China are usually divided into three categories: the first is the independent hospice care institution, which is mostly small and medium size. The service project includes hospice care for hospitalization, hospice care service at home, and day hospice care service. The second is the family hospice care: the patient lives in his own home, the family members provide basic daily care, and hospice care organization provides routine hospice care for patients and family members. The third is the attached hospice care institution, which is the “hospice care ward” set up in hospitals, nursing homes, and community health stations. The use of hospice care services in China has increased for over 30 years, Tianjin college established the first hospice care institution in mainland China In July 1988, namely, “palliative care research center”, and the li ka shing foundation founded the first hospice home in Shantou medical university in 1998. Up to December 2017, the foundation has donated more than 630 million RMB, and more than 30 hospitals have been funded to establish hospice homes, which are now distributed in 27 provinces across the country. Medical staff and social workers provide household service to the patient and their family members who meet the “poverty, and pain, home, free” four conditions, and it formed a union with Chinese characteristics service mode.

There needs to be an integrated service approach to try to address death and bereavement. It focuses on the nature of multidimensional problems and the solutions that need to be addressed, which are rooted in fairness and the quality of care for all people. Social work plays an important role in providing hospice care services in multidisciplinary teams. The hospice care movement emphasizes an integrated approach that strives to focus on the personal life experience and lays stress on the importance of multidisciplinary collaboration since the mid-1960s [5]. The purpose of this study is to improve the quality of life of end-of-life care patients. The intervention of social work methods is very important, so it has a very close relationship with social work practice. Death is an essential part of life, so it certainly affects each member of society. The national cancer center released the latest cancer data from China, showing that 4.29 million new cases of cancer are reported annually in China, accounting for 20% of new cases worldwide and 2.82 million deaths, and the elderly, in particular, were 36%. The goal of quality hospice care is not to cure the disease, but to minimize the suffering and pain of the patient [1]. According to the statistics of China cancer registration 2017, the incidence of cancer among the elderly in our country exceeds 75%.

The second is family hospice care, the patient lives in his own home, the family members provide basic daily care, hospice care organization provides routine hospice care for patients and family members; The third is the attached hospice care institution, which is the "hospice care ward" set up in hospitals, nursing homes and community health stations. The use of hospice care services in China has increased for over 30 years , Tianjin college established the first hospice care institution in mainland China In July 1988, namely "palliative care research center", the li ka shing foundation founded the first hospice home in Shantou medical university in 1998. Up to December 2017, the foundation has donated more than 630 million RMB, and more than 30 hospitals have been funded to establish hospice homes, which are now distributed in 27 provinces across the country. Medical staff and social workers provide household service to the patient and their family members who meet the "poverty, and pain, home, free" four conditions, it formed a union with Chinese characteristics service mode.

Social workers are evaluators of patients’ psychological and social problems, psychological supporters of patients and their families, caregivers of patients and their families, integrators and advocates of resources needed by patients’ families, collaborators of medical workers, trainers, and leaders of volunteers. The principles and philosophies of social work have long been regarded as closely related to hospice care, providing a comprehensive holistic care that encompasses respect, dignity, and difference [6, 7]. According to Adams, the definition of empowerment is individuals and groups have the ability to understand their situation, exercise their power, and achieve their own goals, so as to maximize the quality of their own lives and the lives of others. Song liyu, a scholar in Taiwan, believes that empowerment means that individuals have a positive attitude towards their own abilities, consciously control their own lives, and influence the surrounding environment when they need it. Empowerment is the basic theory and practice of social work for the elderly. It affirms individual self-esteem and realizes self-worth. It can give the elderly the initiative in solving their own problems and difficulties, make the dying elderly have positive role cognition, and enhance their ability to choose and control life. Social workers highlight the unique role and core skills in the hospice work. This feature is to pay close attention to the dying person in the family, community, and regional and cultural environment, and attention to hospice care should be a part of daily life for all social workers.

In most cancer hospitals in China, it is very important for the hospice team to cooperate with the patients and their families in their comprehensive evaluation and service. Although multidisciplinary team will also exist problems, such as perspectives of individual members of the team often do not get equal weight, so we need to provide an in-depth research how to achieve quality hospice care through conducting effective teamwork which is to provide patient-centered services.

### Hospice care and social work

Hospice care refers specifically to team-based, patient-centered supportive services to terminally ill patient and their families, including patient pain management, medical health care, and support of social resources and spiritual welfare (http://www.nhpco.org/about/hospice-care).

Although the role of social work varies from service to service, an important aspect of end-of-life care social work includes helping individuals and their families manage losses at every stage of the disease’s trajectory [8]. Social work often works with disadvantaged individuals and groups who are more likely to die prematurely. Therefore, social work plays an important role in end-of-life care and the more general loss experience. Therefore, what social work service needs to do is “support people to live well and die well: establish a social care network framework at the end of life,” recognizes that social care service “mainly occurs in the community environment,” and plays an important role in “promoting supportive communities through extensive community participation” [9].

**Fig. 1:**
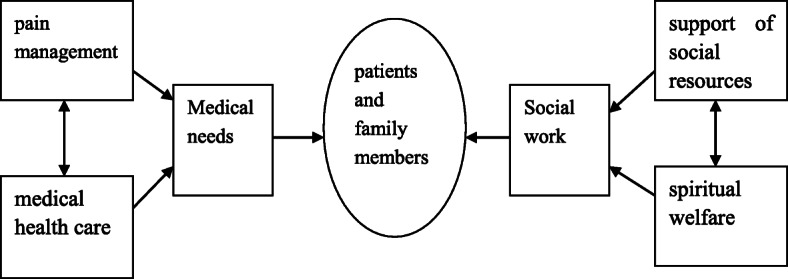
The figure of social work services intervention in hospice care

In China social services and health care services that are integrated together through the authority of the medical social work association, multidisciplinary team of tumor hospital provides continuing health and social care for patients and family members. Hospice care team of tumor hospital achieves holistic family and the whole team and the whole community of five “all” services, it cannot ignore the role of social workers, and social workers need to deal with different environments to provide psychological, social, and spiritual needs care for patients and family members. Through the different care in the form of links, forming quality hospice care lets the patient and family members no matter when and where all can enjoy the professional quality hospice care and obtain more continual care. The figure of social work services intervention in hospice care is shown in Fig. 1.

The relationship in Fig. 1 just shows that through efforts of medical staff and all kinds of practice of social workers to improve the quality of life of patients and their families, there is no such thing as the subjective loss of the right to self-determination. Because first of all, when patients are very conscious, they are willing to make self-decisions, and when the patient is not conscious, it is natural that the trusted family members make decisions. Some patients will feel everything is meaningless when know their inevitable death soon; so, what others think does not matter, let the trusted family members make a decision. What’s more, the patients themselves have the thoughts of “a poor family is hard to give up” and “a hot land is hard to leave” in traditional Chinese culture, so they prefer to die at home. What social workers do is to respect the legitimate and reasonable wishes of patients before dying, maintain the rights and dignity of patients receiving hospice care, and finally let patients die peacefully and comfortably, which is the ultimate goal of hospice care.

Figure 1 mainly focuses on various services and interventions during hospice care, as well as subsequent grief counseling and humanistic care for family members. Welfare is the condition that makes people live a happy life. It includes not only the protection and care that the human body deserves, but also the factors that affect the free development of human intelligence and spirit. Mental welfare is a kind of systematic and long-term psychological service based on people. It aims to help solve the psychological and behavioral problems of patients and their families, so as to maintain people’s mental health and ensures the positive state of subjective feelings. The arrows in Fig. 1 are not causal; the one-headed arrows represent one-way interactions, and the double-headed arrows represent interactions. Professional quality hospice care is conform to the objective requirement of the high quality of life of human beings, by letting patients get quite comfortable at the time of death; when patients died later, let the family do not leave any regret and psychological shadow, and medical workers have been using superb medical treatment and clinical nursing means and scientific psychological care methods that meet their medical needs, social workers needed for the integration of various social resources and building a hospice care team with high quality help them establish correct life values and to achieve peace of mind, calm, and comfortable that can meet the needs of the non-medical. Finally, it promotes the realization of professional quality hospice care by providing patients and their families with spiritual welfare and social resource support.

Most hospice care services are for patients with advanced cancer, and some are critically ill with multiple organ failure. They are patients with no hope of treatment under current medical conditions and expected life span within 6 months. Medical personnel needs to take on the patients’ pain management, sputum suction, pills, pipeline replacement, and so on; in the last period of the health care, the work will be timely communication with their families, to promote the interaction between the patient and family, many of the patient and family also want at the last moment to reduce unnecessary rescue, and finally, the patient in family accompanied died peacefully. Social workers promote the formation of a hospice care service system with multiple subjects and various forms of service. With the goal of “improving the quality of life of dying patients”, social workers actively communicate with patients and their families, carry out spiritual care, and provide various social resources and spiritual welfare. Compared with the care for other common diseases, hospice care requires more professional personnel, such as psychological counselors, nursing workers, and religious guides. Figure 1 shows that the quality of life of patients and their families is improved through medical care and social work intervention, and the interaction and positive communication among members are indirectly promoted through the efforts of medical staff and social workers.

The focus of social workers is the non-medical needs of patients and family members, and their role is to assist patients and family members in coping with the process of cancer and death and to adjust the stress associated with disease and death, to understand the interaction situation between individual family members, to enhance the positive communication between members, to seek the social resources of all parties, and to help them solve problems. After the death of the patient, the social worker needs to provide grief counseling to his family members and help his family members to pass the grieving period safely.

## Methods

Since there is a limited understanding of the practice of end-of-life care in China and the role of social work in it, exploratory research is the most appropriate [10]. Doing the hospice programs itself is a kind of exploratory research, and at the time of the hospice social work project, we reviewed our service experience and the research process and results of the social work of hospice patients and their families. This retrospective case study analysis method has been implemented, namely, “information unit” is determined and reviewed. The case study method can help to describe the intensity and depth of the occurrence to reflect the authenticity and representativeness of the “case.” The purpose of this project is to reflect on the social work service. Through reflection and comment on the work process and results, we use this method of critical extraction to provide important parameters for analyzing the nature and development process of things.

Social workers also have some professional skills and practical training that can help patients and family members address taboo issues around death and bereavement. A core value of social work is to value and focus on community networks as an important source of support for individuals and families. Social workers in end-of-life care services provide models of prevention and intervention: prevention includes access to death and the normalization of bereavement within the community, and intervention includes post-crisis support. This research method of this paper focuses on the development and empowerment of individuals, families, and communities and will make greater contributions to social services.

Semi-structured interview is an open interview method that allows the diffusion of ideas along with the interview. During the interview, both sides are likely to have new ideas on the other’s questions and answers. The study area is Taiyuan, Shanxi Province, China. The interview is conducted in a quiet and independent space without being disturbed. The purpose and significance of the interview will be introduced before the interview. In order to obtain information on palliative care for elderly cancer patients, this paper conducted 27 interviews with 2 medical personnel, 10 nursing personnel, 3 social worker, 6 family caregiver, and 6 patients, of whom 72% were female and 28% were male, with an average age of 57.6 years old and + 15.2 years old (30–81 years old). In terms of caregiver roles, 46% were wives, 36% were husbands, 22% were sons, 4% were siblings, and 2% were daughters-in-law. In nursing education, 22% had a primary school degree, 28% had a junior high school degree, 36% had a high school degree, and 14% had a college degree. A total of 26% of caregivers stopped working to care for relatives, 30% retired, 26% stayed at home, and 18% continued to work. The main contents of the interview include the knowledge reserve of hospice care for medical staff and the dilemma of hospice care practice: patients’ physiological comfort, psychological satisfaction, sense of security, social interpersonal relationship, spiritual care, community management, and spiritual belief. During the interview, the researchers used a recorder to record the non-verbal information such as facial expressions and body movements. The interview ended with a summative inquiry, asking “I have nothing more to ask, do you have anything to say.” After obtaining the consent of the interviewees, this part of the interview will be translated into the interview text for subsequent analysis. Within 24 h after the interview, the recording file was converted into a document. For privacy and ethical reasons, the recording of the interview was deleted after it was confirmed that it was no longer used. Colaizzi data analysis method was used to analyze the data. The specific steps were as follows: first, carefully read the interview records and extract the significant statements; secondly, the author codes the recurring viewpoints and collects the encoded viewpoints to write a detailed description; and third, identify similar viewpoints and return to the research object for verification.

My experience of hospice social work practice in China has been through involvement in the care of patients in Taiyuan cancer hospital. In the service process, there is a highly cooperative team, which includes doctors, nurses, and social workers, and doctors hold the core positions in the team. However, during the service process, he also listens to the reasonable suggestions of social workers, and it is very important to have a highly equal team cooperative relationship during the project operation. The following typical cases are taken from my practice in Taiyuan cancer hospital, and they graphically highlight this point.

### Elderly cases of hospice care in Taiyuan cancer hospital

#### Case study 1

Mr. Z was an old Chinese man who claims to have an undiagnosed lung problem. Investigative surgery revealed terminal lung cancer, and he cannot be operated on. The patient received the services of li ka shing’s hospice care project, so he and his family are well aware of the severity of the disease. When we visited the house, we found the patient lying in bed, without saying a word. The patient’s wife did not have a formal job and lived on odd jobs. Tragically, their son was born in 1994 with a genetic birth defect called phenylketonuria, which led to mental impairment with no self-care ability. The daughter of the patient went to the university in Shanghai, and because of her father and brother’s illness, she had to leave school to help her mother take care of her father and brother. Health care would be provided by his wife, and doctors, nurses, and social workers began to communicate with his wife, asking about the patient’s recent illness and psychological condition.

This case provides an important basis for the urgent improvement of communication between different professionals in the hospital, in order to develop targeted plans that take into account the needs of patients and family members. From the perspective of social work value, the patient’s right to self-determination is not fully implemented, and the patient’s physical and mental state determines that he cannot make rational decisions rationally. In the context of traditional Chinese culture, the old man has no right to choose when he is dying. Instead, he has family members to choose for him. This may differ from the western “human rights” social work values. Confucianism in traditional Chinese culture is the ideological foundation of the Chinese people. According to the Confucian theory of death attribution, death is a natural destination, and it is a painful release for the dying patients who are suffering from pain. The patient may have become irrational at the end of his life because of the pain, and he has complete trust in his family. Family members also expect patients to die peacefully, comfortably, and with dignity when medical resources fail to treat them.

In this case, the patient has lost the confidence of all kinds of life, basically in the state of waiting for death, and the family members decide all kinds of things in the family, which makes the social workers think that they are in a dilemma. This ethical dilemma is the subjective loss of the right to self-determination.

#### Case study 2

Ms. Fan is a patient with advanced uterine cancer. A few months ago, I came into contact with Ms. Fan because she accepted the hospice program of our li ka shing foundation. She is now in terminal cancer and needs hospice care. She has severe leg edema, urinary incontinence, and bedsore and is now confined to bed. Her husband now offers nursing care, and he has an elderly mother to look after. After my enquiry, I established that her family was impoverished (although still ineligible for government financial assistance), and there were few resources in the area that could provide assistance. The elderly mother also has arthritis and relies on painkillers for years to ease her pain. So, her husband is under a lot of psychological pressure, which leads to emotional depression. The patient has not made any consideration and arrangement for herself. The most important concern is the arthritis of the old mother and the single problem of the son.

This case reflects the Confucian family culture of the Chinese people, which values human relations more than nature. For families, the burden of such end-of-life care is still heavy, both financially and in the care of elderly patients. The health care system has tried to adopt a more primary care focus by funding a series of diversionary programs to move services back to the community and to better serve elderly cancer patients to reduce their over-reliance on end-of-life care services and families.

The patient has a very optimistic attitude towards her life, which is a state of natural acceptance, but she has great expectations for the future of her family members. As medical social workers play a very important role of integral part of care, it is necessary to comprehensively evaluate her various needs from the perspective of the patient, so as to not only guarantee her life quality physically, but also meet her expectation of life psychologically.

## Results

The results show that there are several problems. First, the medical staff’s knowledge reserve of hospice care is insufficient, and they are faced with practical difficulties. They report that hospice knowledge comes from classroom and experience, and they seldom attend relevant training. A2: “Knowledge of end-of-life care is acquired from the classroom or from teaching materials.” A3: “Experience is acquired from clinical practice and there is no specific method of psychological comfort and care for those near death.” Secondly, the physical and psychological care services for patients. The 21 respondents were more concerned about the patients’ physical pain and psychological maladjustment. A6: “pay attention to the physiological pain and treatment plan of the patient, pay attention to the changes of the condition in time, and inform the doctor for treatment in time if there is any danger.” A9: “give patients certain physical comfort, such as shaking hands, patting on the back, pay attention to privacy protection, do the most basic nursing work, talk with patients more, give psychological support.” A1: “China's death culture emphasizes ‘the return of fallen leaves to the roots’, so many patients want to die in their own homes, so that they can feel safe.” Thirdly, the care service for patients’ family members. All respondents agreed that grief counseling and continued emotional care were needed until the family members returned to their normal lives. A4: “talk to family members, listen to them carefully, tell them about the prognosis of patients, don't let family members have unrealistic hopes, let family members make rational decisions.” A8: “let the family know the meaning of life and the knowledge of hospice care, and do the follow-up grief counseling. However, due to the special national conditions and culture of China, in order to avoid new conflicts between doctors and patients, this aspect is not professional and sustainable.” Fourth, the disturbance of social work in hospice care service. The contradiction between doctors and patients still exists, there is a lack of death education, the public has no rational concept of death, and the hospice care resources are not in place. A14: “patients near death have no quality of life by any form of rescue measures, but some family members deliberately make trouble in the hospital because they cannot accept the death of their loved ones, resulting in doctor-patient conflict.” A7: “some patients and their families have too high expectations of the current medical level and irrational cognition of death.” A15: “there are altogether three professional hospice care institutions, 20 practicing doctors engaged in hospice care, and only 10 social workers in the hospice care community. It is difficult to recruit volunteers, and it is not long-term and sustainable.”

Hospice care team first needs to make a comprehensive evaluation of the real condition of the patient and family at the time of intervention services, in order to have a basic understanding of her physical symptoms and health plans [11]. Social workers are in a unique position at this stage, and they can help people voluntarily expounded their own pain and various needs and share effective information such as differential diagnosis and self-management with patients before and after medical access. Social workers analyze the real needs of patients and provide targeted and effective services according to the service demand analysis model of the elderly patients under end-of-life care.

**Fig. 2:**
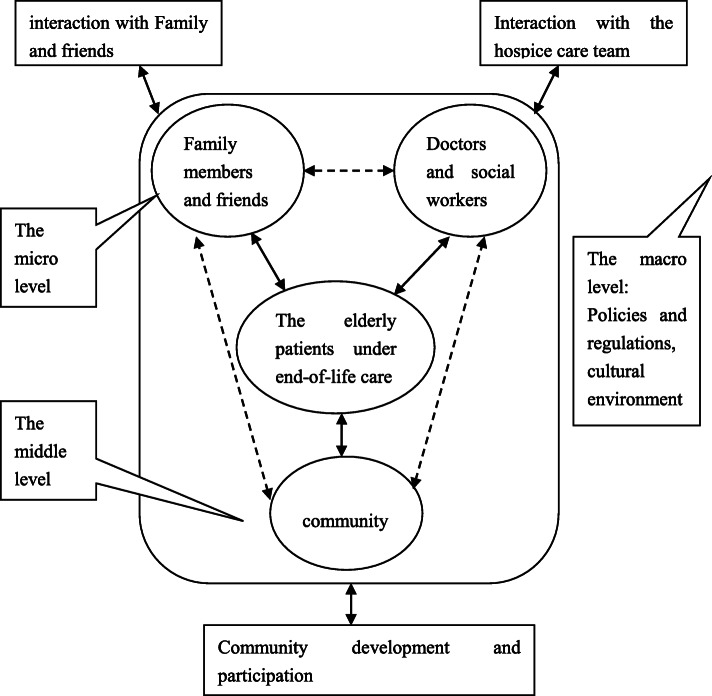
the service demand analysis model of the elderly patients under end-of-life care

Before the formal intervention, hospice team needs to strengthen training and check for individual team members on a regular basis, to answer questions on any intervention, informal assessment of their reactions, and to comply with the degree of intervention model [12]. At the time of an official intervention to patients, need to multiple levels of needs assessment in the first place, including to reduce the pain of physiological needs, the demand for life care, the demand for spiritual care, the demand of community resources, and the need for government preferential policies such as all kinds of needs (Fig. 2).

In the new era, researchers are implementing and testing interventions in different environments to address the need for a combination of treatment and care. This approach requires standardization of the design and measurement of actual cases [13]. End-of-life care patients are more likely than the general population to be diagnosed with life-threatening diseases, and all have experienced intensive medical interventions [14]. Until recently, however, it was not recognized that the traumatic effects of a life-threatening illness or that intensive medical interventions themselves could lead to trauma [9, 15, 16]. EMDR therapy is performed in a short protocol that uses bilateral stimulation (visual, tactile, or auditory) to create a double focus of attention and promote adaptive memory integration, and it can help reduce various medical trauma symptoms [17–19]. This treatment is more suitable for elderly patients with hospice care.

In China, the national health and health commission have vigorously promoted the correct concept of life and death and filial piety and encouraged the provision of high-quality hospice care services for the elderly. Social work departments take the way of mutual assistance to raise funds; under the background of the long-term care insurance system of the ministry of human resources and social security, it provides basic life care and medical care services for the dying elderly. Social workers provide training to staff of institutions providing integrated medical and nursing services. The content includes comprehensive assessment of the characteristics of the dying elderly, family relationship analysis of the elderly, spiritual cognition of the elderly, etc. It can cultivate the concept of comprehensive diagnosis and treatment for the elderly medical staff and improve the quality of life of the dying elderly.

The ministry of civil affairs issued a “geriatric social work service guide.” Social work participation in end-of-life care for the elderly has been specified in detail. Some cities appeared “hospice care + social work+ funeral service” comprehensive social work service mode. Social workers actively guide and standardize the volunteer force to participate in hospice care, strengthening the construction of hospice care social work service supervision team, improve the ability of using social work expertise to carry out end-of-life care, and achieve the effect of high-quality end-of-life care. In this study as shown in Table 1, service classification includes public service, concept service, and rights service. Social work intervention includes resource linkage, correct view of life and death and filial piety, and non-medical resource service. The corresponding effects of end-of-life care include delivering services, building networks and caring for the elderly, correct concepts, solving problems and harmonious relationships, accurate services, humanistic care, and spiritual gratitude.

**Table 1:** Functional system table of social work intervention in elderly patients with hospice care

Service classification	Social work intervention	hospice care effect
public service	Links to resources	Building networks and caring for the elderly
Concept service	Correct view of life and death	Correct ideas and harmonious relationships
Rights service	Non-medical services	Precision service and humanistic care

Community based on the characteristics of the base and advantages, relying on the community workers shard bag piece, the door to visit system, assist residents to establish health files, timely find the elderly in need of end-of-life care, communicate and cooperate with hospital and social workers timely define the service content, strengthen team building, and give full play to the positive role of community hospice care service in this field.

The intervention of social work services centers on the care of the dying elderly. The intervention of social work service centers on the care of patients’ pain, which can help patients reduce their fear and pain, realize their sense of dignity and value, establish harmonious family relations, and solve various problems of end-of-life care with various forces and resources in the social network. Social workers use their professional advantages to help the elderly and their families obtain more comprehensive social welfare.

## Discussion

Our research results show that in the social work intervention in hospice care, intervention study determined the hospice care team three key strategies: (1) provide professional education of hospice care for the patients and family members and external resources, (2) make a comprehensive assessment of medical needs and non-medical needs, design and implement the patient diagnosis and treatment tracking system, record the real-time situation of patients in detail, and (3) build the micro, medium and macro intervention service system in the community. These strategies have proven to be challenging, the substantive issues of hospice care are often vague, there are many inconsistencies in conventional physician-patient hospice care discussions, and patients’ perception of the prognosis of service teams in a community setting is essentially vague. Our research suggests ways to solve these problems.

Patients and family members describe that high-quality end-of-life care in the community environment field is consistent with the values of life and filial piety, which can realize the sense of respect and values of patients, and brings spiritual relaxation and emotional comfort to family members. In some ways, these findings reflect the experience of studying patients and family members receiving hospice care at home in the community. Research suggests that hospice care and community successful cooperation key points include effective cooperation between hospice care team and the community, commitment to service plan and execution is necessary, clearly define the role, the rights, and responsibilities for the elderly hospice team and the community, and to maximize the elderly hospice service quality.

Our research has some limitations. First of all, our findings cannot be extended to all social work services, because the purpose of qualitative research is not to promote, but to provide in-depth insights into exploratory research. Second, our interview did not include palliative care, so there may be a lack of in-depth exploration of more service processes.

## Conclusion

Social work intervention methods have made significant contributions to the work of end-of-life care for the aged, which has great potential to optimize the development of high-quality hospice care services. This includes a correct understanding of death, a dialectical approach to the problems associated with death, and the encouragement of community support and sustainable development of holistic care [20]. This is a professional intervention method that requires the re-establishment of normal service needs and team roles. Its focus is on the participation and authorization of community resources, rather than simply responding to the needs of patients and their families. It encourages the sharing of the expertise and case experience of service team members so as to meet the social care needs of their patients and family members. Social work intervention can avoid the monopoly role of non-medical needs. It has the basis of dialectical philosophical values, professional training, and practical experience of excellent cases, and plays an important and efficient role in providing quality services for the elderly in hospice care.

The primary purpose of this study was to gather the most realistic information about the current situation and problems in end-of-life care and then to address them through social work interventions. In the process of the study, it was found that medical staff lacked sufficient knowledge of hospice care, professional hospice care staff was also very scarce, patients and their families lacked community death education, independent places for spiritual care, and community resources participation was not high. Our study also has limitations. The interviewees were selected by hospices in accordance with the principle of respecting the will, so the representativeness of the research results may not be sufficient. However, we found that through the intervention of social work, the establishment of a multidisciplinary team of hospice care can improve the quality of life of patients and their families and provide them with all-round and multi-angle humanistic care. We believe these findings can provide a good basis for larger or more representative studies in the future.

## Data Availability

Data sharing is not applicable to this article as no datasets were generated or analyzed during the current study.
